# Human DNA helicase HELQ participates in DNA interstrand crosslink tolerance with ATR and RAD51 paralogs

**DOI:** 10.1038/ncomms3338

**Published:** 2013-09-04

**Authors:** Kei-ichi Takata, Shelley Reh, Junya Tomida, Maria D. Person, Richard D. Wood

**Affiliations:** 1 Department of Molecular Carcinogenesis, The University of Texas MD Anderson Cancer Center Science Park, Smithville, TX 78957, USA; 2 ICMB Protein and Metabolite Analysis Facility, University of Texas at Austin, Austin, TX 78712, USA; 3 Graduate School of Biomedical Sciences at Houston, Houston, TX 77030, USA

## Abstract

Mammalian HELQ is a 3′–5′ DNA helicase with strand displacement activity. Here we show that HELQ participates in a pathway of resistance to DNA interstrand crosslinks (ICLs). Genetic disruption of *HELQ* in human cells enhances cellular sensitivity and chromosome radial formation by the ICL-inducing agent mitomycin C (MMC). A significant fraction of MMC sensitivity is independent of the Fanconi anaemia pathway. Sister chromatid exchange frequency and sensitivity to UV radiation or topoisomerase inhibitors is unaltered. Proteomic analysis reveals that HELQ is associated with the RAD51 paralogs RAD51B/C/D and XRCC2, and with the DNA damage-responsive kinase ATR. After treatment with MMC, reduced phosphorylation of the ATR substrate CHK1 occurs in *HELQ*-knockout cells, and accumulation of G_2_/M cells is reduced. The results indicate that HELQ operates in an arm of DNA repair and signalling in response to ICL. Further, the association with RAD51 paralogs suggests HELQ as a candidate ovarian cancer gene.

## Abstract (web summary)

Agents that cause DNA interstrand crosslinks are widely used to treat cancer. Takata *et al.* show that the DNA helicase HELQ associates with ATR and RAD51 paralogs, which are components of DNA repair pathways, and helps defend human cells against agents that induce DNA interstrand crosslinks.

## Graphical abstract

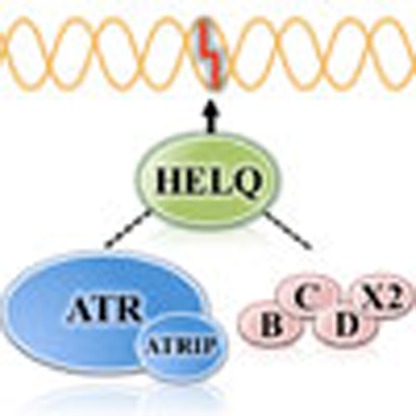

DNA interstrand crosslinks (ICLs) are particularly toxic because they disrupt genetic information on both strands, potently inhibiting DNA replication and transcription. DNA ICLs are produced by many environmental agents including photoactivatable furocoumarins present in some plants, aldehydes from industrial sources and endogenous agents, including products of lipid peroxidation and nitric oxide^1^,^2^. Compounds that form ICLs are widely used in cancer chemotherapy and include platinum-based compounds, mitomycin C (MMC), psoralens, nitrogen mustards and derivatives (melphalan and chlorambucil) and nitrosoureas such as BCNU^1^,^2^. Multiple pathways are implicated in various regulated steps of ICL repair, including many proteins associated with pathways of homologous recombination, the Fanconi anaemia (FA) network, mismatch repair, nucleotide excision repair and translesion DNA synthesis^3^,^4^,^5^,^6^,^7^,^8^.

The RAD51 paralogs (RAD51B, RAD51C, RAD51D, XRCC2 and XRCC3) are a group of related proteins with distant similarity to the RAD51 protein, which mediates homologous DNA pairing during recombination. Inactivation of any RAD51 paralog confers substantial cellular sensitivity to agents causing ICL^9^,^10^. However, it has been difficult to obtain mechanistic insights into how the RAD51 paralogs operate in ICL repair^10^,^11^. Models for the role of the RAD51 paralogs are diverse and range from loading of RAD51 on DNA^5^,^12^ to post synaptic functions in RAD51 filament dissociation^13^,^14^, to direct involvement in Holliday junction resolution^15^. In fact, there may be several functions for the paralogs. Furthermore, protein complexes of subsets of the RAD51 paralogs exist^10^,^11^, but specific functions of these subcomplexes remain unclear.

Helicase-like enzymatic activities are predicted to be useful for several steps in ICL repair. Two of the FA proteins, BRIP1 (also known as FANCJ) and FANCM contain conserved helicase motifs and can translocate on DNA in an ATP-dependent fashion^5^. However, specific roles for these and other helicases in ICL repair have yet to be identified.

The *HELQ* gene, originally designated *HEL308* (ref. 16), is a candidate for participation in ICL repair. HELQ is a DNA helicase that translocates in the 3′–5′ direction and can displace oligonucleotides of 70 nt or more from DNA^16^. The homologue from *Solfolubus solfataricus* is powerful enough to readily overcome a streptavidin–biotin interaction^17^. On the basis of model substrates *in vitro*, it has been proposed that HELQ opens up the parental strands at a blocked DNA replication fork and remodels nascent lagging strand intermediates to facilitate the loading of subsequent factors required for DNA damage processing or restart of DNA replication^18^. However, the *in vivo* function of mammalian HELQ remains to be explored.

Here we report that genetic disruption of *HELQ* enhances the sensitivity of human cells to ICL-inducing agents, including MMC. The enhanced sensitivity is independent of the FA pathway. Sister chromatid exchange (SCE) frequency and sensitivity to UV radiation or topoisomerase inhibitors is unaltered. Analysis of interacting protein partners revealed that HELQ is associated with the RAD51 paralogs RAD51B/C/D and XRCC2, and with the DNA damage-responsive kinase ATR. Reduced phosphorylation of the ATR substrate CHK1 occurs in *HELQ*-knockout cells treated with MMC, and accumulation of G_2_/M cells is reduced. The results indicate that HELQ operates in DNA repair and cell cycle checkpoint signalling in response to ICL. The association with RAD51 paralogs and the frequent copy number loss of HELQ in ovarian cancers suggests *HELQ* as a candidate ovarian cancer predisposition gene.

## Results

### Inactivation of HELQ sensitizes human cells to ICL damage

**Figure 1:**
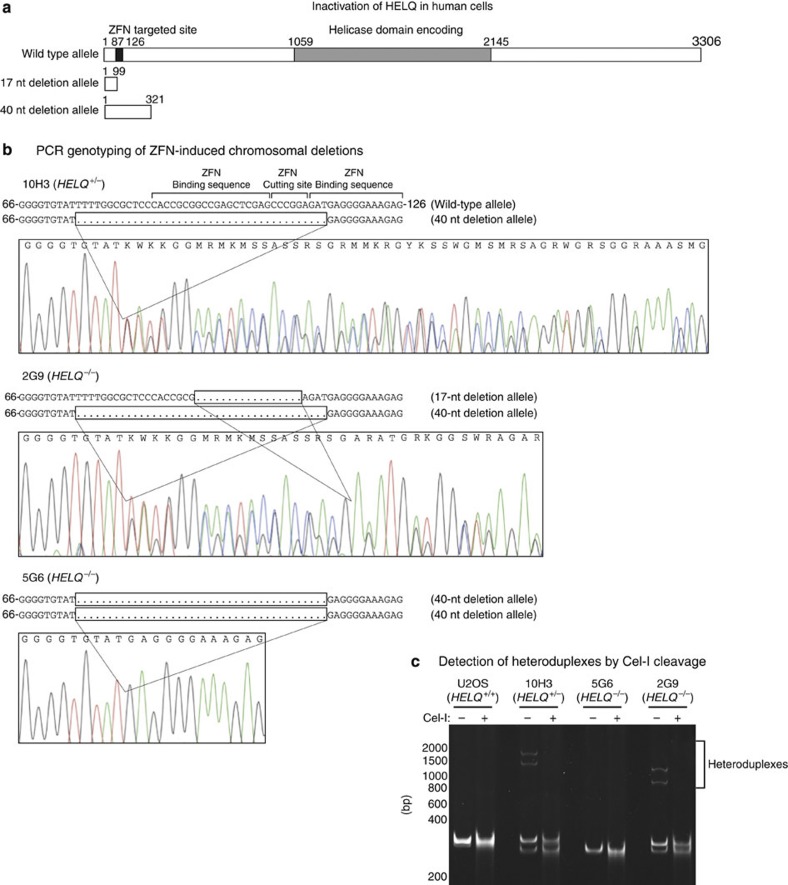
Inactivation of HELQ in human cells. (**a**) Both copies of HELQ in the human osteosarcoma cell line U2OS were sequentially targeted in the first exon by ZFNs, producing deletions that yield early truncating frameshifts. A HELQ heterozygous knockout cell line (10H3) was established with a 40-nt deletion in one allele, changing the open reading frame after amino acid 24, with a stop codon arising after 106 total amino acids. Retargeting established the HELQ disrupted cell lines 5G6 with a second 40-nt deletion and 2G9 with a 17-nt deletion in the other allele at amino acid 31, creating a stop codon after 32 amino acids. The open reading frame of the wild-type allele is 3306, bp, 17-nt deletion allele 99 bp and 40-nt deletion allele 321 bp. (**b**) Sequence chromatograms of PCR products for 10H3, 5G6 and 2G9 showing the deletions. Nucleobases G, C, A, T are colour-coded black, blue, green, and red, respectively. The *HELQ-*specific ZFNs-binding sites and cutting site are shown on the wild-type allele. (**c**) Polyacrylamide gel electrophoresis result of PCR products for each cell line showing allele specific amplification as well as the formation of heteroduplexes in 10H3 and 2G9.

To explore the biological function of HELQ, the human gene was disrupted in the U2OS osteosarcoma cell line by targeting the first exon with specific zinc finger nucleases (ZFNs). A heterozygous knockout (*HELQ*^+/−^) cell line (10H3) was isolated, and two knockout (*HELQ*^−/−^) cell lines (5G6 and 2G9) were obtained by retargeting 10H3 with the same ZFNs. The ZFN disruptions introduced frameshift mutations early in the open reading frame (Fig. 1).

**Figure 2:**
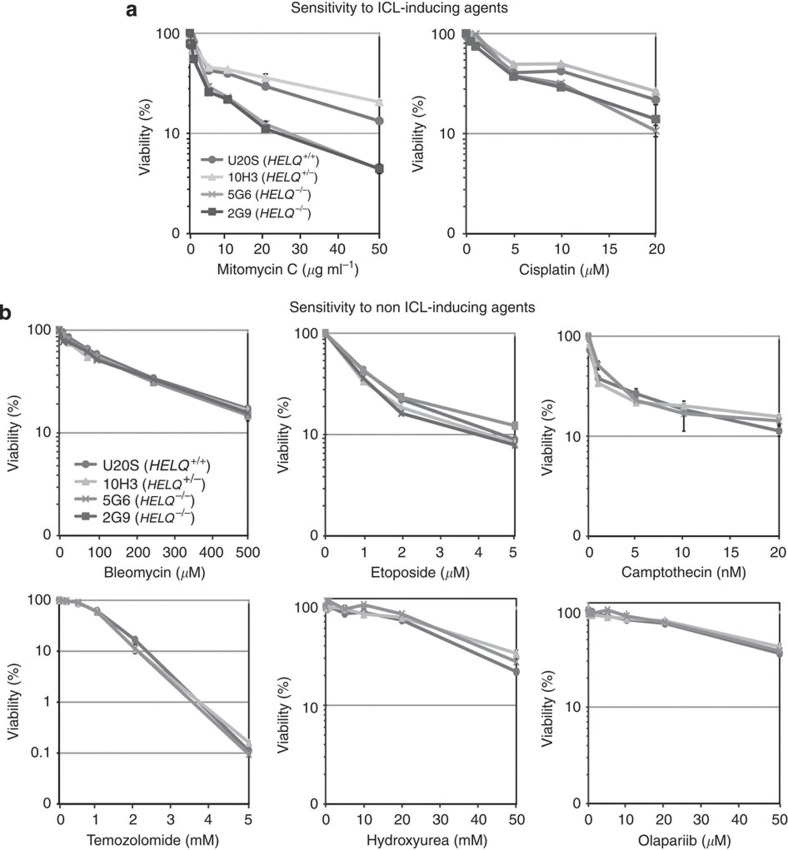
HELQ-knockout cell lines are sensitive to ICL-inducing agents. The inactivation of HELQ sensitizes U2OS cells to the DNA ICL-inducing agents, MMC and cisplatin (**a**) but not to non-ICL-inducing agents bleomycin, etoposide, camptothecin, temozolomide, hydroxyurea and olaparib (**b**). Cells were exposed to indicated doses of MMC for 24 h, cisplatin for 24 h, bleomycin for 48 h, etoposide for 48 h, camptothecin for 48 h, temozolomide for 48 h, hydroxyurea for 24 h and olaparib for 48 h. Viability was determined by measuring ATP content as described in Materials and Methods. *HELQ*^+/+^ U2OS (circles), 10H3 *HELQ*^+/−^ (grey triangles), 5G6 *HELQ*^−/−^ (dark grey cross) and 2G9 *HELQ*^−/−^ (black squares). The mean of three separately plated and treated wells is shown, with s.d. indicated by error bars. The hypersensitivity of 5G6 compared with USOS and 10H3 was confirmed in independent experiments for MMC (three experiments) and cisplatin (two experiments).

HELQ-deficient cells were examined for sensitivity to treatment with DNA-damaging agents in comparison with wild-type and heterozygous cells. The HELQ-deficient U2OS cells were sensitive to the ICL-inducing agents MMC and cisplatin (Fig. 2a and Supplementary Fig. S1A), but were not consistently sensitized to bleomycin, etoposide, camptothecin, temozolomide, hydroxyurea, olaparib or UV irradiation (Fig. 2b and Supplementary Fig. S1B). This suggests a specific function for HELQ in the processing of ICL. Further, MMC-induced radial chromosome formation was moderately increased in *HELQ*^−/−^ cells (Supplementary Fig. S1C). Radial chromosome formation after treatment with ICL-inducing agents is a cytological hallmark of FA cells with defects in ICL processing^19^.

### HELQ and FA pathway ICL sensitivity are additive

**Figure 3:**
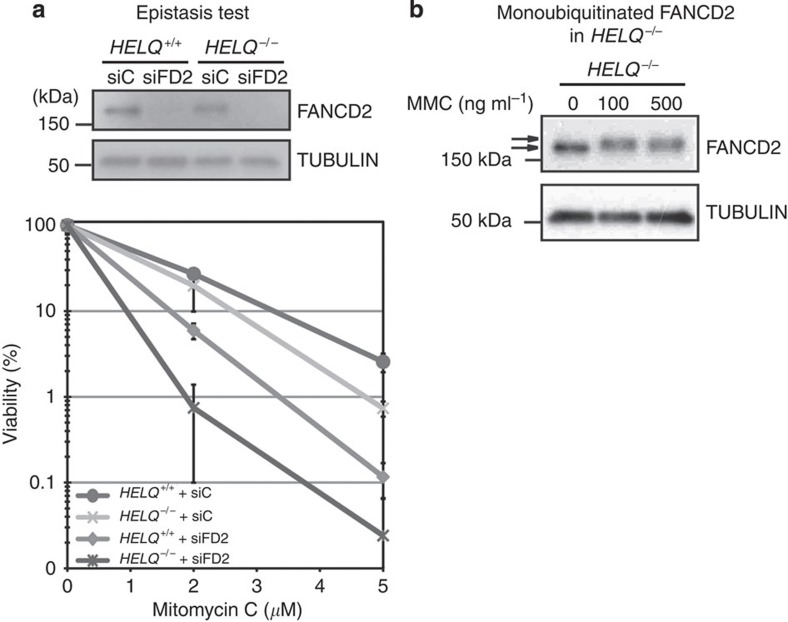
Genetic relationship between HELQ and FANCD2. (**a**) Upper panel: immunoblot showing efficacy of siRNA-mediated knockdown of FANCD2 (siFD2) in *HELQ*^+/+^ U2OS and 5G6 (*HELQ*^−/−^) cells. siC served as a negative control and α-tubulin as loading control. Lower panel: cell survival determined by using clonogenic survival assays. FANCD2-depleted *HELQ*^−/−^ cells were the most sensitive to MMC, and were more sensitive than mock-depleted *HELQ*^−/−^ or FANCD2-depleted *HELQ*^+/+^ U2OS cells. The mean of four separately plated and treated plates is shown, with s.d. indicated by error bars. (**b**) Immunoblot showing status of mono-ubiquitinated FANCD2 (upper arrows) in *HELQ*^+/+^ U2OS and *HELQ*^−/−^ cells is incubated with indicated amount of MMC for 24 h. Full-size immunoblots can be found in Supplementary Fig. S5.

To test whether the function of HELQ in ICL processing is dependent on the FA pathway, we used short interfering RNA (siRNA) knockdown of *FANCD2* gene expression in wild-type and *HELQ*^−/−^ cells. *FANCD2*-knockdown cells were more sensitive than *HELQ*^−/−^ cells. A knockdown of *FANCD2* in *HELQ*^−/−^ cells resulted in even higher sensitivity to MMC (Fig. 3a). HELQ therefore appears to have a role in ICL processing that is separate from the FA pathway. Consistent with this notion, the canonical FANCD2 monoubiquitination response to MMC treatment was still observed in *HELQ*^−/−^ cells (Fig. 3b).

### HELQ interacts with the BCDX2 complex and ATR

**Figure 4:**
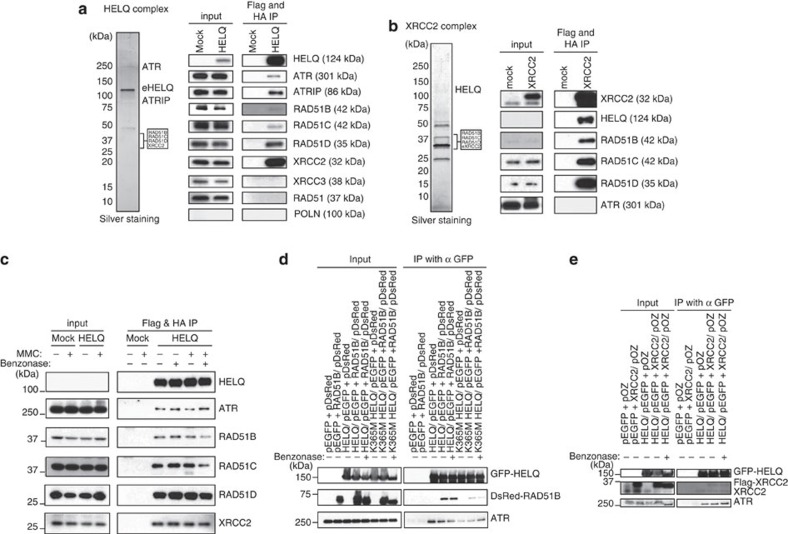
HELQ interacts with the BCDX2 RAD51 paralog complex and ATR. (**a** and **b**) The HELQ and XRCC2 complexes were immunopurified from nuclear extracts prepared from HeLa S3 cells expressing FLAG-HA-epitope-tagged HELQ and XRCC2, respectively. The complexes were sequentially purified with anti-FLAG and anti-HA antibodies. The complexes were resolved by SDS-PAGE on a 4–20% gradient gel and visualized by silver staining. Approximate migration positions of proteins identified in gel sections are shown (left). Immunoblotting with specific antibodies confirmed the presence of the proteins in the HELQ complex (right). (**c**) BCDX2 and ATR associate with HELQ before and after MMC treatment. The FLAG-HA-tagged HELQ complex was purified from HeLa S3 with or without MMC treatment for 18 h. Similar amounts of RAD51B, C, D, XRCC2 and ATR were coimmunoprecipitated with HELQ before and after MMC treatment. Nucleic acid digestion with Benzonase did not influence HELQ-BCDX2 and HELQ-ATR associations. (**d**) GFP-tagged HELQ and DsRed-Monomer-tagged RAD51B were transiently coexpressed in HEK293T cells. The whole-cell extracts prepared from those transfected cells were sonicated and incubated in the presence or absence of Benzonase. GFP-tagged HELQ was immunoprecipitated with anti-GFP antibody and DsRed-Monomer-tagged RAD51B, and endogenous ATR in the immunoprecipitated samples were detected with anti-RAD51B and anti-ATR antibodies. (**e**) GFP-tagged HELQ and flag-tagged XRCC2 were transiently coexpressed in HEK293T cells. HELQ and XRCC2 association was examined with XRCC2-specific antibody after immunoprecipitation of GFP-tagged HELQ with anti-GFP antibody. Flag-tagged XRCC2 (upper bands) and endogenous XRCC2 (lower bands) were both coimmunoprecipitated with GFP-tagged HELQ. Full-size immunoblots can be found in Supplementary Fig. S5.

To determine molecular pathways relevant to HELQ, we searched for associated proteins *in vivo*. HELQ was stably expressed as a Flag and HA epitope fusion (eHELQ) in HeLa S3 cells. eHELQ was purified from the nuclear extract by immunoprecipitation sequentially with anti-Flag and anti-HA antibodies^20^,^21^ (Fig. 4a). The HELQ complex was separated on a gradient gel and proteins from gel sections were identified by liquid chromatography–mass spectrometry. With high-ranking confidence, we identified components of the ‘BCDX2 complex’ consisting of RAD51B, RAD51C, RAD51D and XRCC2. The DNA damage-responsive kinase ATR and its partner ATRIP were also identified as binding partners (Supplementary Table S1). In a control Flag-HA purification from HeLa S3 cells transfected with empty control vector, neither BCDX2 nor ATR was present. Further, we performed parallel experiments in the same system using Flag-HA-tagged POLN^22^,^23^ as bait, and detected neither RAD51 paralogs nor ATR.

There are five RAD51 paralogs that form two distinct subcomplexes in mammalian cells, denoted as BCDX2 and CX3 (composed of RAD51C and XRCC3)^9^,^24^. All members of the BCDX2 subcomplex were detected, but no XRCC3 was identified in HELQ coimmunoprecipitates, indicating the absence of CX3. The presence of these proteins in the HELQ complex was confirmed by immunoblotting with specific antibodies (Fig. 4a).

To independently test the association of HELQ with the BCDX2 complex, similar experiments were performed to capture proteins associating with XRCC2. XRCC2 was stably expressed as a Flag and HA epitope fusion (eXRCC2) in HeLa S3 cells. The eXRCC2 was sequentially immunoprecipitated with anti-FLAG and anti-HA antibodies and examined by silver staining and mass spectrometry (Fig. 4b). The most abundant specific proteins in this XRCC2 complex as indicated by the number of unique peptides were XRCC2 (25), HELQ (47), RAD51D (23), RAD51B (22) and RAD51C (18) (Supplementary Table S2). RAD51B, RAD51C, RAD51D and HELQ were verified by immunoblotting to be present in the XRCC2 complex (Fig. 4b). This also represents the first detection of endogenous HELQ in human cells. Neither the eHELQ or eXRCC2 complexes included RAD51, or DNA polymerase POLN, previously proposed as a HELQ-interacting partner^25^ (Supplementary Table S1).

### HELQ interactions persist in cells exposed to MMC

We then asked whether HELQ-BCDX2 and HELQ-ATR interactions persist in cells treated with MMC. Flag and HA epitope tagged HELQ (eHELQ) was purified from nuclear extracts of cells treated without MMC or with MMC for 18 h. Similar amounts of ATR and BCDX2 were coimmunoprecipitated with HELQ before and after MMC treatment (Fig. 4c). These interactions were sustained after incubation with the DNase/RNase Benzonase, indicating that the HELQ complex can be isolated in the absence of intact polynucleotides (Fig. 4c).

To confirm these interactions in another cell line, and to exclude an epitope-tag-specific interaction, green fluorescent protein (GFP)-tagged HELQ was coexpressed with DsRed-Monomer-tagged RAD51B in HEK293T cells. After immunoprecipitation with GFP antibody from whole-cell extract, DsRed-Monomer-tagged RAD51B and endogenous ATR were identified, in the presence or absence of Benzonase. The GFP-tag alone did not interact with DsRed-Monomer-tagged RAD51B (Fig. 4d). A K365M mutation in the Walker A consensus ATP-binding site of HELQ lacks DNA helicase activity^16^. HELQ K365M retained the ability to interact with ATR and DsRed-Monomer-tagged RAD51B (Fig. 4d). The interaction between GFP-tagged HELQ and DsRed-Monomer-tagged RAD51B was also confirmed in U2OS cells (Supplementary Fig. S2). We also coexpressed GFP-tagged HELQ and Flag-tagged XRCC2 in HEK293T cells, and confirmed that those proteins were coimmunoprecipitated with anti-GFP antibody. Moreover, endogenous ATR and XRCC2 were coimmunoprecipitated with GFP-tagged HELQ (Fig. 4e).

**Figure 5:**
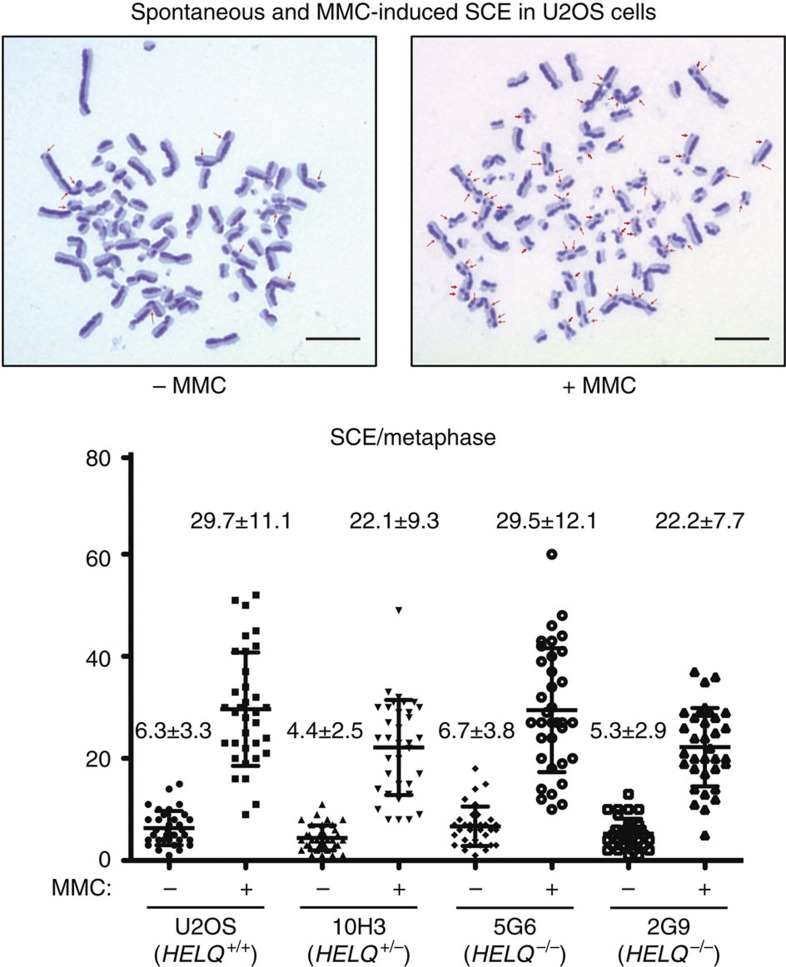
Sister chromatid exchange frequency in HELQ-knockout cells. Cells were labelled with BrdU during two cell cycle periods with or without MMC treatment (15 ng ml^−1^ for 32 h). Spontaneous and MMC-induced SCEs in 35 metaphase cells for each sample were counted. Scatter plots show the frequency of cells with the indicated numbers of SCEs per cell. The mean number of SCEs per cell±the s.d. is shown on the top of each plot. Arrows indicate visible SCEs. Scale bars: 10 μm.

It has been proposed that RAD51 paralogs may have a role in facilitating RAD51 assembly at the sites of DSBs and promoting intersister chromatid recombinational repair^9^. However, HELQ disruption did not affect MMC-induced RAD51 foci formation (Supplementary Fig. S4A). Further, neither spontaneous nor MMC-induced SCE frequencies were affected by *HELQ* disruption (Fig. 5). The lack of reduction of spontaneous SCE frequency is consistent with results obtained for RAD51 paralog disruption in rodent cells, which have normal or only modestly reduced frequencies of spontaneous SCE^26^,^27^,^28^. However, damage-induced SCE is reduced in *Rad51d*-knockout MEFs^29^.

### HELQ has a role in promoting CHK1 activation

**Figure 6:**
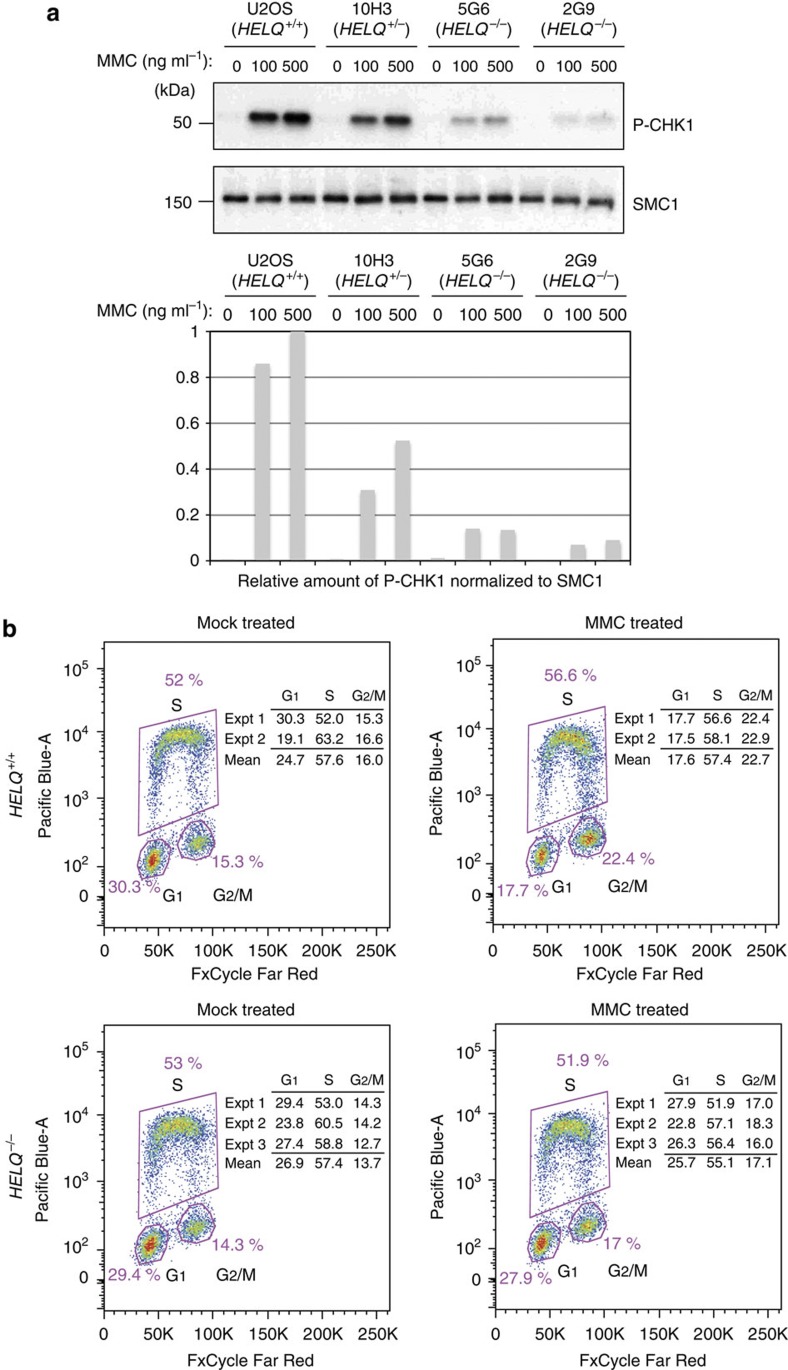
HELQ is required for CHK1 activation. (**a**) Ser345 phosphorylation of CHK1 in U2OS and HELQ mutant cells (10H3; *HELQ*^+/−^, 5G6 and 2G9; *HELQ*^−/−^) collected 24 h after exposing to an indicated amount of MMC. SMC1 was used as a loading control. (**b**) G_2_/M populations in U2OS and *HELQ*^−/−^ U2OS cells in response to MMC treatment. *HELQ*^−/−^ and *HELQ*^+/+^ U2OS cells were exposed to 30 ng ml^−1^ MMC for 1 h and then collected at 24 h. The cells were pulse-labelled with EdU before collecting, fixed and stained. Cell cycle distributions were determined by flow cytometry and presented as the mean of two independent experiments (Expt) 1 and 2 for U2OS and three independent experiments 1, 2 and 3 for *HELQ*^−/−^ U2OS cells (Expt 1 and 2 for 5G6 and Expt 3 for 2G9). The figure illustrates data for Expt 1 (see also Supplementary Figure S3). Full-size immunoblots can be found in Supplementary Fig. S5.

ATR kinase activity is triggered during ICL repair; the mechanism of this activation is complex and actively studied^30^,^31^. As *HELQ*^−/−^ cells were sensitive to DNA-crosslinking agents and HELQ interacts with ATR, we asked whether HELQ status influences activation of the ATR-mediated DNA damage checkpoint after MMC treatment. To this end, U2OS (*HELQ*^+/+^), 10H3 (*HELQ*^+/−^), 5G6 (*HELQ*^−/−^) and 2G9 (*HELQ*^−/−^) cells were incubated with MMC. Using CHK1 Ser345 phosphorylation as a marker, we found that MMC-induced CHK1 phosphorylation was significantly reduced in *HELQ*^−/−^ cells (Fig. 6a).

CHK1 phosphorylation signals to arrest mammalian cells in the G_2_/M phase of the cell cycle after DNA damage by MMC^32^,^33^. To measure G_2_/M arrest, cells were incubated either in the absence or presence of MMC, and their cell cycle profile was analysed. Twenty-four hours after MMC treatment, *HELQ*^−/−^ cells had a lower G_2_/M population (17.1%) than wild-type (22.7%) (Fig. 6b). This finding was confirmed by an independent analysis method (Supplementary Fig. S3). The results indicate that HELQ has a role in the activation of G_2_/M arrest after MMC treatment.

On the basis of model substrates *in vitro*, it has been proposed that HELQ opens up the parental strands at a blocked DNA replication fork and remodels nascent lagging strand intermediates to facilitate the loading of subsequent factors required for DNA damage processing or restart of DNA replication^18^. At a DNA lesion present on only one DNA strand (such as aUV radiation-induced cyclobutane pyrimidine dimer), DNA replication forks are stalled and single-stranded DNA (ssDNA) is generated, which is required for ATR activation^34^. We examined global formation of RPA foci after treatment of growing cells with MMC, and found that it was not significantly affected in *HELQ*-knockout cells (Supplementary Fig. S4B). Generation of ssDNA at replication forks blocked at monoadducts may not be the function of HELQ that promotes CHK1 phosphorylation. This is consistent with the unchanged sensitivity of HELQ-deficient cells to UV radiation or strand-breaking agents. However, when a DNA replication fork stalls at an ICL, processing to generate ssDNA may be impaired, and HELQ may have a more specific function in this context. A global assay of ssDNA formation would not detect such a defect, as a maximum of only about 5% of the adducts caused by MMC can be ICLs^35^.

## Discussion

This study reveals that HELQ is involved in cellular resistance to ICLs in human cells, and places HELQ within a complex associated with the RAD51 paralogs. This is consistent with the cellular sensitivity to agents causing ICLs, which is conferred by loss of RAD51 paralogs^9^,^10^. The specific role of the RAD51 paralogs remains elusive, and it has been difficult to obtain mechanistic insights into how they operate in ICL repair^5^,^10^,^11^. We found that human HELQ only interacts with one of the two RAD51 paralog subcomplexes, BCDX2, whereas the ‘CX3 complex’ involving RAD51C and XRCC3 is not tightly associated. BCDX2 and CX3 may have independent functions in DNA repair. In chicken DT40 cells, a double RAD51B RAD51D mutant is no more sensitive to cisplatin than RAD51B single mutants, whereas an XRCC3 RAD51D mutation affecting both subcomplexes had an additive effect on the sensitivity^36^. Our findings indicate that HELQ has a BCDX2-related function affecting sensitivity to ICLs.

The unchanged SCE frequency in HELQ-defective cells does not rule out a role for HELQ (or the RAD51 paralogs) in homologous recombination. SCE are thought to arise via homologous recombination to overcome DNA replication blocks. As previously suggested^27^,^37^, restart of stalled replication forks by homologous recombination may occur primarily by a noncrossover mode of Holliday junction resolution that does not produce SCE as cytologically identifiable events.

An association of HELQ and RAD51 paralogs is consistent with the phenotypic similarity of several egg-shell morphology mutants of *Drosophila*^38^. Mus301, a *Drosophila* homologue of HELQ, was isolated as a ‘spindle-shaped’ egg-shell morphology mutant called *spn-C*. Other similar spindle morphology mutants with female sterility include *spn-A* (Rad51) and the two *Drosophila* Rad51 paralogs, *spn-B* (Xrcc3-like) and *spn-D* (Rad51C-like) as well as *okra* (Rad54)^39^. In *Caenorhabditis elegans*, the *HELQ* homologue is termed *helq-1* and its disruption confers sensitivity to ICL-inducing agents^40^. Apparently HELQ-1 together with RFS-1, the *C. elegans* RAD51 paralog, aids in the disassembly of RAD51 filaments from double-stranded DNA during meiosis^14^,^41^. The normal frequency of loading of RAD51 suggests a possible function for HELQ following RAD51 filament assembly and strand invasion in homologous recombination. A late role for some mammalian RAD51 paralogs in recombination has also been proposed, perhaps in resolution or disassembly of the recombination machinery^13^.

The RAD51 paralogs also have connections with a group of FA-related proteins, which are involved in repair and tolerance of ICL^42^. The HELQ-associated protein RAD51C is tentatively designated as FANCO^43^, and a homozygous *XRCC2* mutation is present in an FA patient^44^. In the present experiments, HELQ was not epistatic to the downstream target of the FANC core complex, FANCD2 (Fig. 3a), and FANCD2 was not identified in the HELQ complex (Supplementary Table S1). Other FA-related pathway proteins are not epistatic with the FA pathway in ICL repair, including SLX4 (also known as FANCP) and FAN1 (FANCD2/FANCI-associated nuclease 1)^45^,^46^,^47^. HELQ is a candidate FA-related gene, but like SLX4 and FAN1, HELQ may have functions that only partially overlap with the FA pathway.

ATR is involved in both CHK1 phosphorylation and FANCD2 mono-ubiquitination^48^, but those pathways can proceed independently^49^. Although RAD17 and TOPBP1 are required for ATR-mediated CHK1 phosphorylation, chicken cells lacking RAD17 or human cells treated with siRNA-targeting TOPBP1 are able to sustain FANCD2 monoubiquitination^31^,^49^. FANCD2 was ubiquitinated after MMC treatment in *HELQ*-knockout cells (Fig. 3b). This indicates that HELQ is involved in ATR-mediated CHK1 activation but not in ATR-mediated FANCD2 monoubiquitination. A direct HELQ-ATR association may be important for CHK1 phosphorylation. The helicase activity-deficient HELQ K365M mutant was still immunoprecipitated with ATR *in vivo* (Fig. 4d). ATR was identified in the HELQ complex but not in the XRCC2 complex, which suggests that the association of HELQ with ATR does not require the BCDX2 complex (Fig. 4a,b).

**Figure 7:**
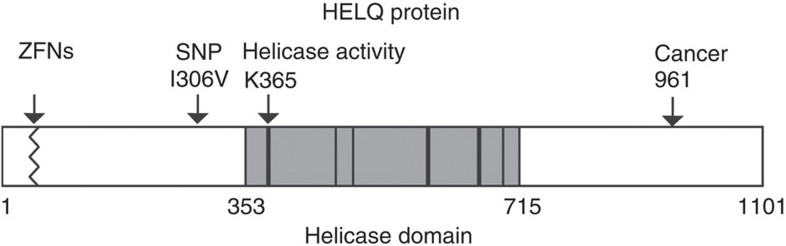
Human HELQ protein variants. The helicase domain is shaded with conserved motifs shown as bars. Lysine K365 mutated in this study is highly conserved in the ‘Walker A’ site of helicase motif 1. The ZFNs disruption introduces truncating frameshift mutations after amino acid number 24 or 31 (see Fig. 1). Copy number loss in serous ovarian cancer is summarized in Table 1. Mutations in *HELQ* have been found in various sequenced cancers from TCGA but their phenotypic effect is uncertain. For example, one colorectal cancer (TCGA-AG-A002) harbours a homozygous mutation truncating the protein at residue 961, which is C-terminal of the conserved helicase motifs. Recent genome-wide association studies for susceptibility to upper aerodigestive tract cancers (head and neck cancers) pinpointed a variant in *HELQ*. In one study, the polymorphism rs1494961 in *HELQ* was found as one of the two top hits modifying the association between smoking pack-years and head and neck squamous carcinomas^57^. This same variant was found in another study as one of five most significantly associated with upper aerodigestive tract cancers^58^. This single-nucleotide polymorphism would give rise to Val or Ile at residue 306. The higher risk T/T allele encodes Ile. This amino-acid alteration lies outside the conserved DNA helicase motifs and either Ile, Asn, or Met is found at this position in other vertebrate sequences.

The connection described here between HELQ and the RAD51 paralogs suggests that *HELQ* is a candidate gene for loss or mutation in sporadic or inherited ovarian cancer. Germ line mutations predisposing to ovarian cancer have been found in the *RAD51C*^50^,^51^,^52^,^53^ and *RAD51D* genes^54^. Current data from The Cancer Genome Atlas (TCGA) shows that the frequency of heterozygous loss of *HELQ* in ovarian cancer is comparable to *RAD51C* and *RAD51D* (Table 1). There are currently seven serous ovarian cancer patient samples listed with *HELQ* homozygous deletion, which is more than found for the RAD51 paralogs, except *RAD51D* (Table 1). Several other mutations and polymorphisms in *HELQ* have been identified in various sequenced cancers (Fig. 7). A common intronic polymorphism in *HELQ* was recently identified as a locus significantly associated with age at natural menopause^55^. It will be worthwhile to determine the functional effect of this polymorphism on ovarian recombination function. The capacity for homologous recombination must be sustained in order to maintain human ovarian function with age^56^, indicating that alterations in *HELQ* may be relevant to both cancer and aging.

## Methods

Experimental procedures for cell culture, antibodies, plasmid constructs, mass spectrometry and immunofluorescence are in the supplementary methods.

### ZFN-mediated gene targeting

CompoZr Custom ZFNs designed to target exon 1 of *HELQ* were purchased from Sigma-Aldrich. pZFN1 and pZFN2 plasmids encoding ZFNs-targeting HELQ were transfected to U2OS by Amaxa Cell Line Nucleofector Kit V with programme X-001. The HELQ-specific ZFN binding (upper case letters) and cutting site (lower case) was 5′-CACCGCGGCCGAGCTCGTGCCCGGAGATGAGGGGAAAGAG-3′. The protocol to deliver ZFNs for the gene knockout was provided by Sigma-Aldrich. After the transfection, single-cell clones were sorted into 96-well plates by flow cytometry. Genomic DNA isolated from individual clones was amplified by PCR with ZFN primers F (5′-TTCCATATGGGCTTAGCGTC-3′) and R (5′-GTCCGGGTTTGTATCACCAC-3′), and then each PCR product was used for the Cel-I Nuclease Mismatch assay (Surveyor). Targeted genomic DNA sequence was confirmed after TA-cloning of each Cel-I-positive PCR product. Confirmed clones (heterozygous knockout clones) were isolated and retargeted with the same *HELQ*-specific ZFNs to target the second allele of *HELQ*. The targeted genomic DNA sequence of *HELQ*-knockout cell lines was confirmed by direct sequencing of PCR products amplified with ZFN primers F and R, and sequencing after TA-cloning of the PCR products.

### Survival experiments

For the ATPlite assay (PerkinElmer), 1,250 cells were dispensed per well into white 96-well plates and incubated 24 h before inducing DNA damage. The cells were incubated with DNA damage-inducing agents for the indicated time. After incubation, the cells were immediately lysed and assayed for ATPlite luminescence as described in the manufacturer’s instructions. For the clonogenic assay, 1.0 × 10^5^ cells were plated in 60-mm culture plates and incubated for 24 h before DNA damage induction. Groups of plates were exposed to indicated doses of MMC for 1 h or UVC. After making a dilution series for each group, cells were returned to the incubator until colonies could be detected in the samples (7 to 14 days), and then were fixed, stained and scored for survival.

### Affinity purification of HELQ and XRCC2 complexes

HeLa S3 cells stably expressing FLAG-HA epitope-tagged HELQ (eHELQ) or XRCC2 (eXRCC2) were grown to 1.0 × 10^6^ cells per ml as 9 or 12 l of suspension cultures^20^,^21^. For the MMC treatment, MMC (final 100 ng ml^−1^) was added to the suspension culture 18 h before collection. The supernatant, nuclear extract and chromatin fractions were prepared from the cells, and the HELQ and XRCC2 complexes were immunoprecipitated from the nuclear extracts by incubating with M2 anti-FLAG agarose gel for 4 h with rotation in the presence or absence of 50 U ml^−1^ Benzonase Nuclease (Novagen). After an extensive wash with buffer 0.1B (100 mM KCl, 20 mM Tris-HCl (pH 8.0), 5 mM MgCl_2_, 10% glycerol, 1 mM PMSF, 0.1% Tween-20, 10 mM *β*-mercaptoethanol), the bound proteins were eluted from M2 agarose by incubation for 60 min with 0.2 mg ml^−1^ of FLAG peptide (Sigma-Aldrich) in the same buffer. A total of 100 μl of FLAG antibody-immunoprecipitated material was further purified by immunoprecipitation with protein A sepharose (Pharmacia) conjugated with anti-HA 12CA5 antibody. The bound proteins were washed with 0.1B and eluted with 17 μl of 0.1 M Glycine-HCl (pH 2.5). After the elution, pH was neutralized with 3 μl of 1 M Tris-HCl (pH 8.0). To verify all proteins found in each complex by immunoblotting, only one gel was used per complex. Each membrane was cut horizontally into sections to immunoblot specifically for proteins of various sizes. Owing to the size similarity of certain proteins, such as RAD51B, RAD51C, RAD51D and XRCC2, some membrane sections were stripped using Thermo Restore Western Blot Stripping Buffer and reblotted multiple times for subsequent protein identification.

### Immunoprecipitation assay

HEK293T (2.4 × 10^6^) or 1.0 × 10^6^ U2OS cells were plated in 10-cm plates 24 h before transfection. HELQ/pEGFP-C1 or empty pEGFP-C1 was co-transfected with RAD51B/pDsRed-Monomer-Hyg-C1, empty pDsRed-Monomer-Hyg-C1, XRCC2/pOZC or empty pOZC into the cells with lipofectamine 2000 (Invitrogen). The cells were incubated with the plasmids for 24 h, washed and replaced with fresh medium. Forty-eight hours after transfection, the cells were harvested, frozen in liquid nitrogen and stored at −80 °C. Each cell pellet was suspended with 300 μl of 0.5B (500 mM KCl, 20 mM Tris-HCl (pH 8.0), 5 mM MgCl_2_, 10% glycerol, 1 mM PMSF, 0.1% Tween-20, 10 mM β-mercaptoethanol), frozen in liquid nitrogen, thawed on ice and sonicated. After centrifugation, 900 μl of 2B (40 mM Tris-HCl (pH 8.0), 20% glycerol, 0.4 mM EDTA, 0.2% Tween-20) was added to the supernatant and incubated with 10 μl of anti-GFP agarose for 4 h at 4 °C in the presence or absence of 50 U ml^−1^ Benzonase Nuclease (Novagen). The bound proteins were washed with 700 μl of 0.1B three times and eluted with 30 μl of 2 × SDS-loading buffer (100 mM Tris-HCl (pH 6.8), 4% SDS, 0.2% bromophenol blue, 20% glycerol and 200 mM dithiothreitol).

### siRNA Transfection

The FANCD2-specific Stealth RNAs (designated ‘siFD2’, 5′-AAUGAACGCUCUUUAGCAGACAUGG-3′) (Invitrogen) and ON-TARGETplus Non-Targeting siRNAs as a negative control designated ‘siC’ (Thermo Scientific) were used. The siRNAs were introduced into wild-type or HELQ-knockout U2OS cells. Twenty-four hours before transfection, cells were plated in a six-well plate at 2.0 × 10^5^ cells per well. For each well, 5 pmol of siRNAs was diluted into 250 μl of Opti-MEM (Invitrogen). In a separate tube, 5 μl of Lipofectamine RNAiMAX reagent (Invitrogen) was diluted into 250 μl of Opti-MEM and incubated at room temperature for 10 min. The Lipofectamine RNAiMAX dilution was added into the diluted siRNA duplex and incubated at room temperature for 20 min. Before the transfection, the medium was replaced with fresh 2.5 ml of DMEM supplemented with 10% foetal bovine serum for each well. The Lipofectamine RNAiMAX-siRNA complex was added dropwise to the cells and incubated at 37 °C. After 24 h, the cells were washed, trypsinized and plated with fresh DMEM medium supplemented with 10% foetal bovine serum and 1% penicillin-streptomycin (Invitrogen). To measure the levels of proteins, whole-cell crude extracts were prepared 48 h after the RNA transfection and analysed using immunoblotting. To analyse MMC sensitivity, the cells were treated with the indicated amount of MMC 48 h after the transfection.

### Checkpoint analysis

Twenty-four hours before MMC treatment, cells were plated in six-well plates at 1.5 × 10^5^ cells per well. Then cells were treated with MMC for 24 h and collected with 300 μl of 2 × SDS sample buffer (100 mM Tris-Cl (pH 6.8), 4% SDS; electrophoresis grade, 0.2% bromophenol blue, 20% glycerol, 200 mM dithiothreitol). Fourteen microlitres of each sample/lane was separated on a 4–20% gradient gel and examined by immunoblotting. Band densities were quantified using National Institutes of Health (NIH) image J software.

### Cell cycle analysis

Cells were treated with or without 30 ng ml^−1^ of MMC for 1 h and incubated with fresh medium for 24 or 48 h, collected and stained with FxCycle Far Red (Life Technologies) for 30 min. To determine the proportions of cells in each stage of the cell cycle, cells were pulse-labelled with 10 mM 5-ethynyl-2′-deoxyuridine (Life Technologies) for 1.5 h and collected for staining with Pacific Blue dye for 30 min before FxCycle Far Red staining. During fluorescence-activated cell sorting analysis, EdU-positive cells in S phase were counted and the corresponding percentage in the total cell population was calculated.

### SCE analysis

Cells (1 × 10^5^) were plated in a 10 cm dish. At 24 h, the cells were treated with 0 or 15 ng ml^−1^ of MMC. At 32 h post MMC treatment, the cells were washed with fresh medium and treated with 10 μM 5-bromo-2'-deoxyuridine (BrdU). At 44 h post BrdU treatment, 14.6 g ml^−1^ colcemid was added to the culture. The cells were trypsinized, rinsed with PBS, exposed to 0.075  M KCl for 15  min at 37 °C, fixed in 3:1 methanol:glacial acetic acid at 48 h post BrdU treatment and spread on glass slides. The cells were stained with 0.5 × SSC containing 2 μg ml^−1^ Hoechst 33258 at 3 to 7 days after spreading cells. The cells were then washed with 2 × SSC and 0.5 × SSC, exposed to UVA light (Sylvania 15W F15T8/350BL) at a distance of 10 cm for 1 h without drying the cells and incubated with 0.5 × SSC at 60 °C for 10 min. The cells were Giemsa stained, and 35 metaphases per sample were analysed using a BX41 Olympus microscope with × 100 oil objective and analysed to identify SCE number in each metaphase.

## Author contributions

K.-i.T. together with S.R., J.T. and R.D.W. conceived and designed the study. K-I.T. and S.R. established *HELQ*-knockout cell lines and identified their phenotype. K.-i.T. and J.T. purified HELQ and XRCC2 complexes. M.D.P. designed the mass spectrometry experimental workflow and performed mass spectrometric analysis. K.-i.T. and S.R. performed immunoblotting and immunofluorescence studies. R.D.W. Analysed mutations and polymorphisms of *HELQ* in various sequenced cancers. K.-i.T. and R.D.W. composed the manuscript. All authors discussed the results and commented on the manuscript.

## Additional information

**How to cite this article:** Takata, K. -i. *et al.* Human DNA helicase HELQ participates in DNA interstrand crosslink tolerance with ATR and RAD51 paralogs. *Nat. Commun.* 4:2338 doi: 10.1038/ncomms3338 (2013).

## Supplementary Material

Supplementary Information Supplementary Figures S1-S5, Supplementary Tables S1-S2, Supplementary Methods and Supplementary References

## Figures and Tables

**Table 1:** Copy number variations for *HELQ* and *RAD51* paralogs in ovarian cancer.

**Gene**	**Diploid**	**Heterozygous loss**	**Homozygous loss**	**Gain**	**Amplification**
*HELQ*	174	339 (60%)	7	47	2
*RAD51D*	68	469 (82%)	9	23	0
*RAD51C*	175	271 (48%)	1	114	8
*RAD51B*	200	286 (50%)	3	67	13
*XRCC2*	206	62 (11%)	3	232	66
*XRCC3*	219	210 (37%)	1	116	23

Copy number levels for each gene are shown for 569 samples of ovarian serous cystadenocarcinoma from The Cancer Genome Atlas (TCGA) as accessed through the cbio portal on July 5 2013^59^. These putative gene/patient copy numbers were derived by the GISTIC 2.0 algorithm (Genomic Identification of Significant Targets in Cancer)^60^.
