# Supplementary material for: Human DNA helicase HELQ participates in DNA interstrand crosslink tolerance with ATR and RAD51 paralogs
Source: Nat Commun. 2013 Sep 4;4:2338. doi: 10.1038/ncomms3338 (PMC3778836; doi:10.1038/ncomms3338)
Supplement: Supplementary Information — Supplementary Figures S1-S5, Supplementary Tables S1-S2, Supplementary Methods and Supplementary References [file ncomms3338-s1.pdf]

SUPPLEMENTARY INFORMATION

Human DNA helicase HELQ participates in DNA interstrand crosslink tolerance with ATR and RAD51 paralogs

Kei-ichi Takata, Shelley Reh, Junya Tomida, Maria D. Person, and Richard D. Wood

Supplementary Figure S1

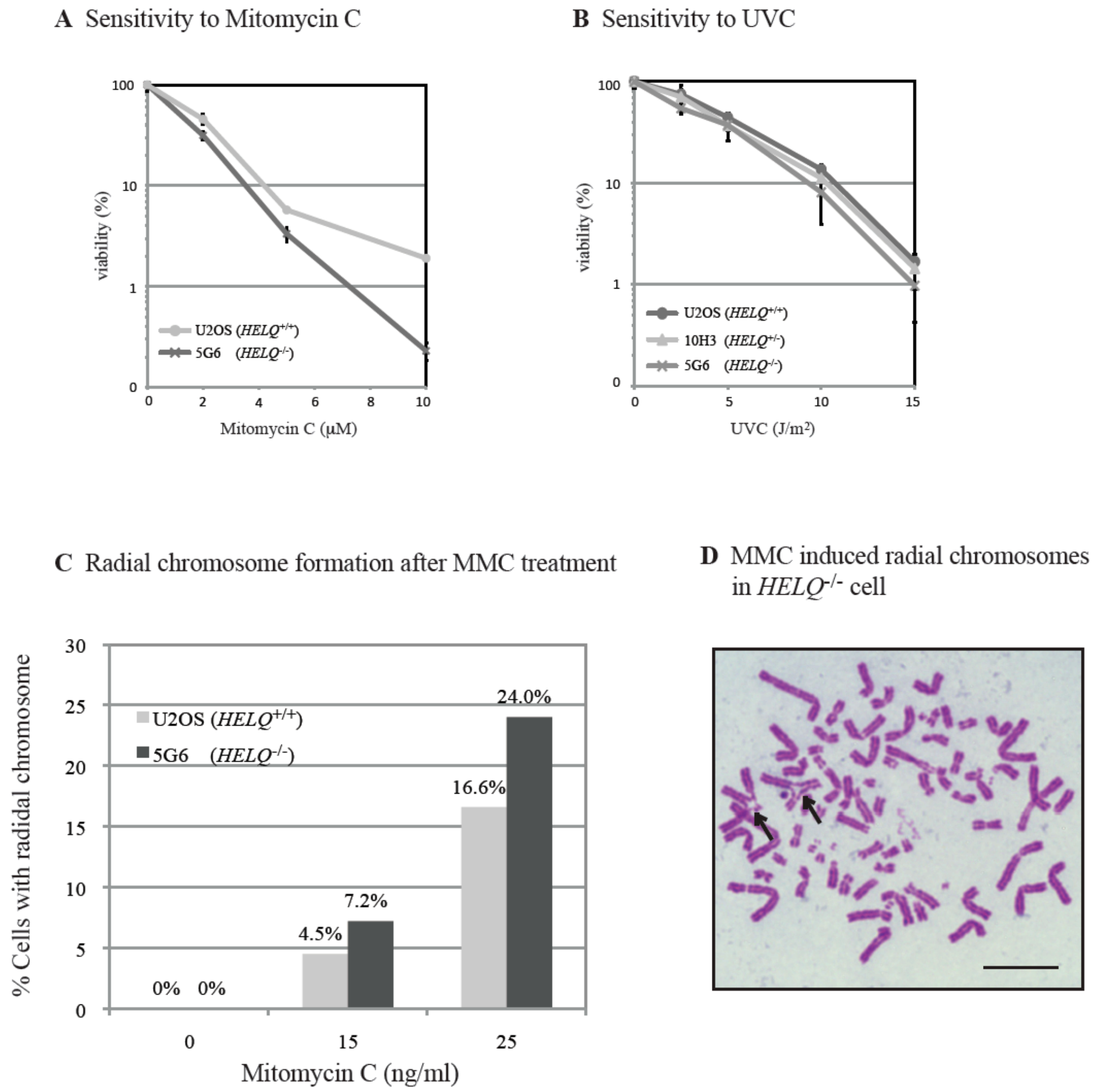

Supplementary Figure S1: ***HELQ* knockout cells are sensitive to MMC.**

Clonogenic cell survival assay determined that the *HELQ*$^{-/-}$ cell line (5G6) showed more sensitivity to MMC than wild type (A) and 5G6 and *HELQ*$^{+/-}$ cell line (10H3) did not show increased sensitivity to UVC (B), the mean results of four separately plated and treated plates for each dose in one experiment is shown, with standard deviation indicated by error bars. (C) MMC-induced radial chromosomes in wild type and 5G6 (*HELQ*$^{-/-}$) cells. Cells were exposed to indicated doses of mitomycin C for 48 hr. 150 metaphases per sample were analyzed. (D) Radial chromosomes in *HELQ*$^{-/-}$ cells exposed to MMC (25 ng/ml for 48 hr). Arrows indicate visible radial chromosomes. Scale bar, 10 μm.

Supplementary Figure S2

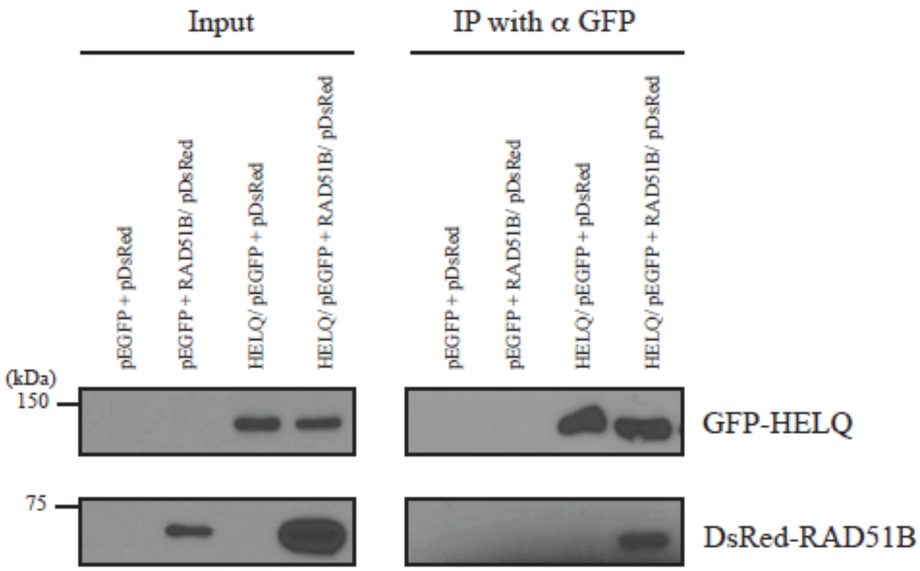

Supplementary Figure S2: **HELQ interacts with RAD51B in U2OS cells.**

GFP-tagged HELQ and DsRed-Monomer-tagged RAD51B were transiently co-expressed in U2OS cells. The whole cell extracts prepared from those transfected cells were sonicated and incubated in the presence or absence of Benzonase. GFP-tagged HELQ was immunoprecipitated with anti GFP antibody, and DsRed-Monomer-tagged RAD51B in the immunoprecipitated samples was detected with anti RAD51B antibody.

Supplementary Figure S3

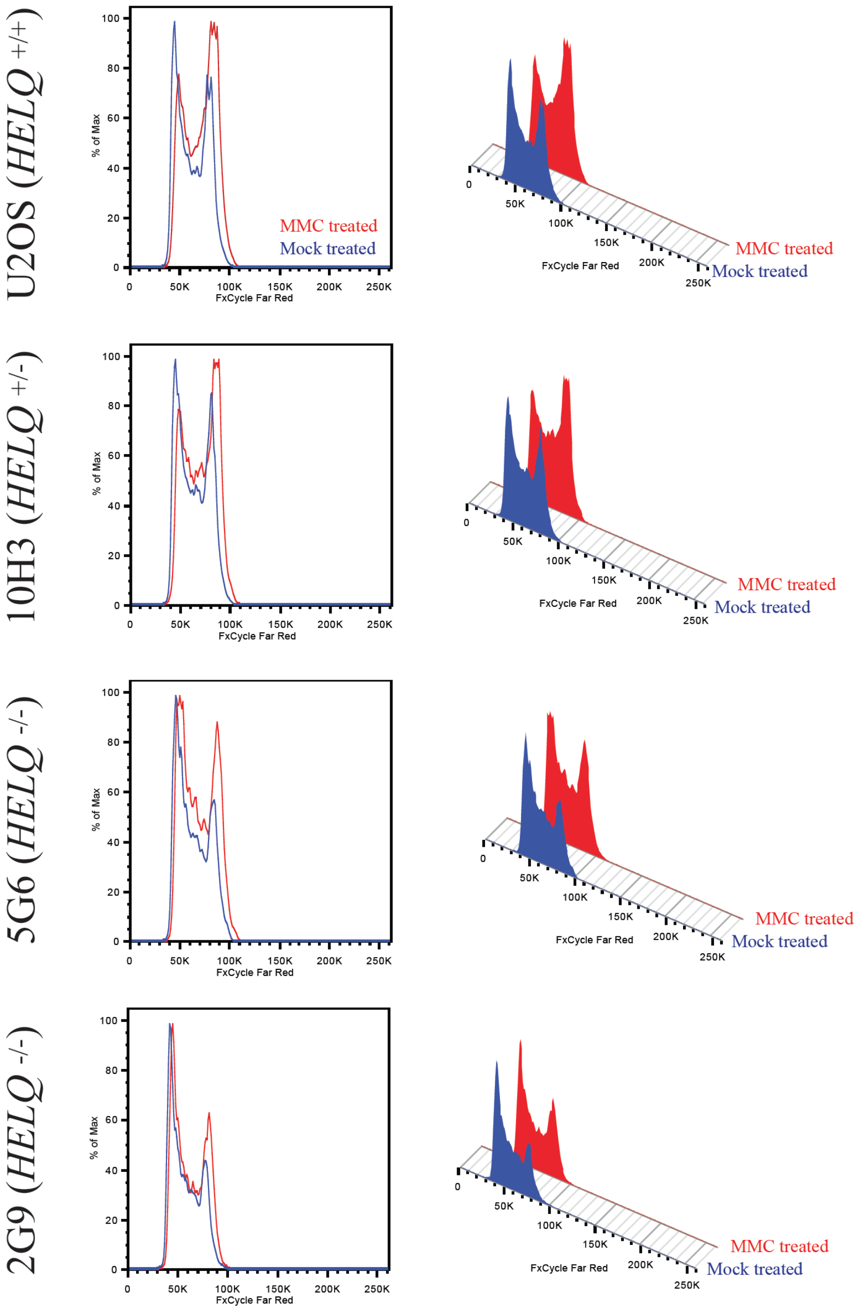

G2/M population in mock or MMC treated cells

	Mock treated	MMC treated
U2OS (*HELQ* $^{+/+}$)	24.2 ± 0.1	38.7 ± 3.0
10H3 (*HELQ* $^{+/-}$)	26.3 ± 3.1	30.4 ± 0.2
5G6 (*HELQ* $^{-/-}$)	23.5 ± 1.2	24.4 ± 3.3
2G9 (*HELQ* $^{-/-}$)	21.0 ± 0.1	21.2 ± 0.6

Supplementary Figure S3: ***HELQ* knockout cells had a lower G_2/M population.** U2OS (*HELQ*$^{+/+}$), 10H3 (*HELQ*$^{+/-}$), 5G6 (*HELQ*$^{-/-}$), and 2G9 (*HELQ*$^{-/-}$) cells were mock treated or treated with 50 ng/ml MMC for 1 hr and harvested at 24 hr. Cell cycle distribution was determined by flow cytometry with FXCycle staining. After MMC treatment, the G_2/M peak in *HELQ*$^{-/-}$ cells was reduced when compared with wild type or *HELQ*$^{+/-}$ cells. G_2/M populations were determined by using the Watson Pragmatic cell cycle model and presented as the mean ± standard error. Mock treated is shown in blue, and MMC treated in red.

Supplementary Figure S4

A

MMC induced RAD51 foci in *HELQ* $^{-/-}$ cells

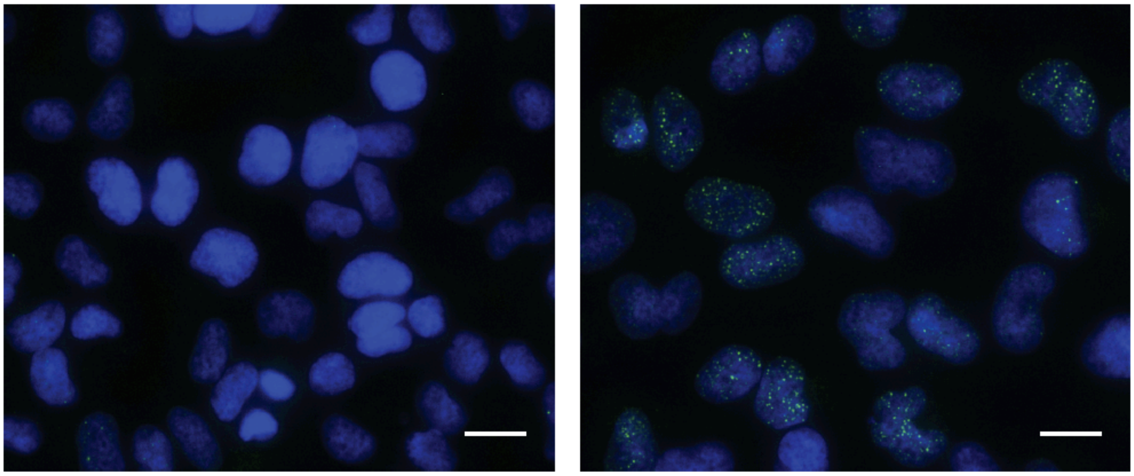

- MMC + MMC

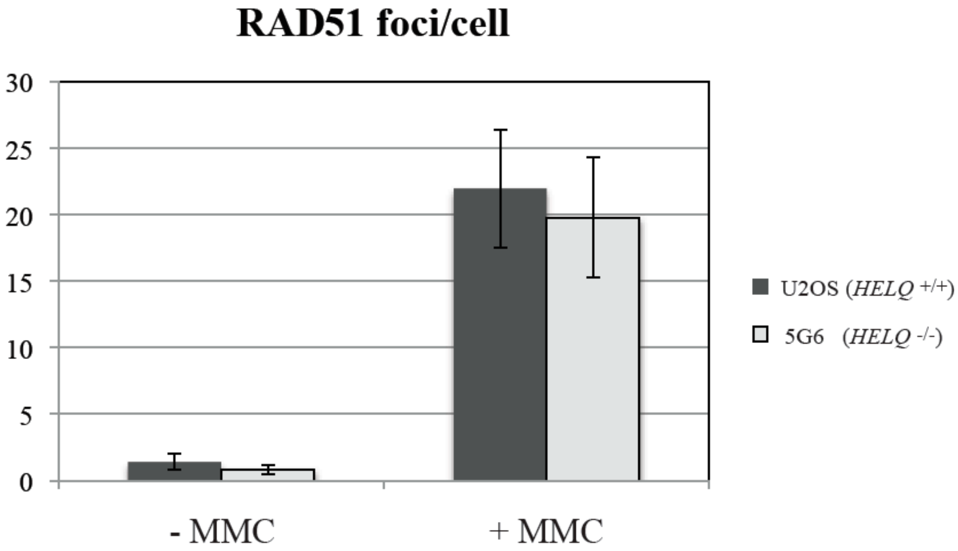

B

MMC induced RPA foci in *HELQ* $^{-/-}$ cells

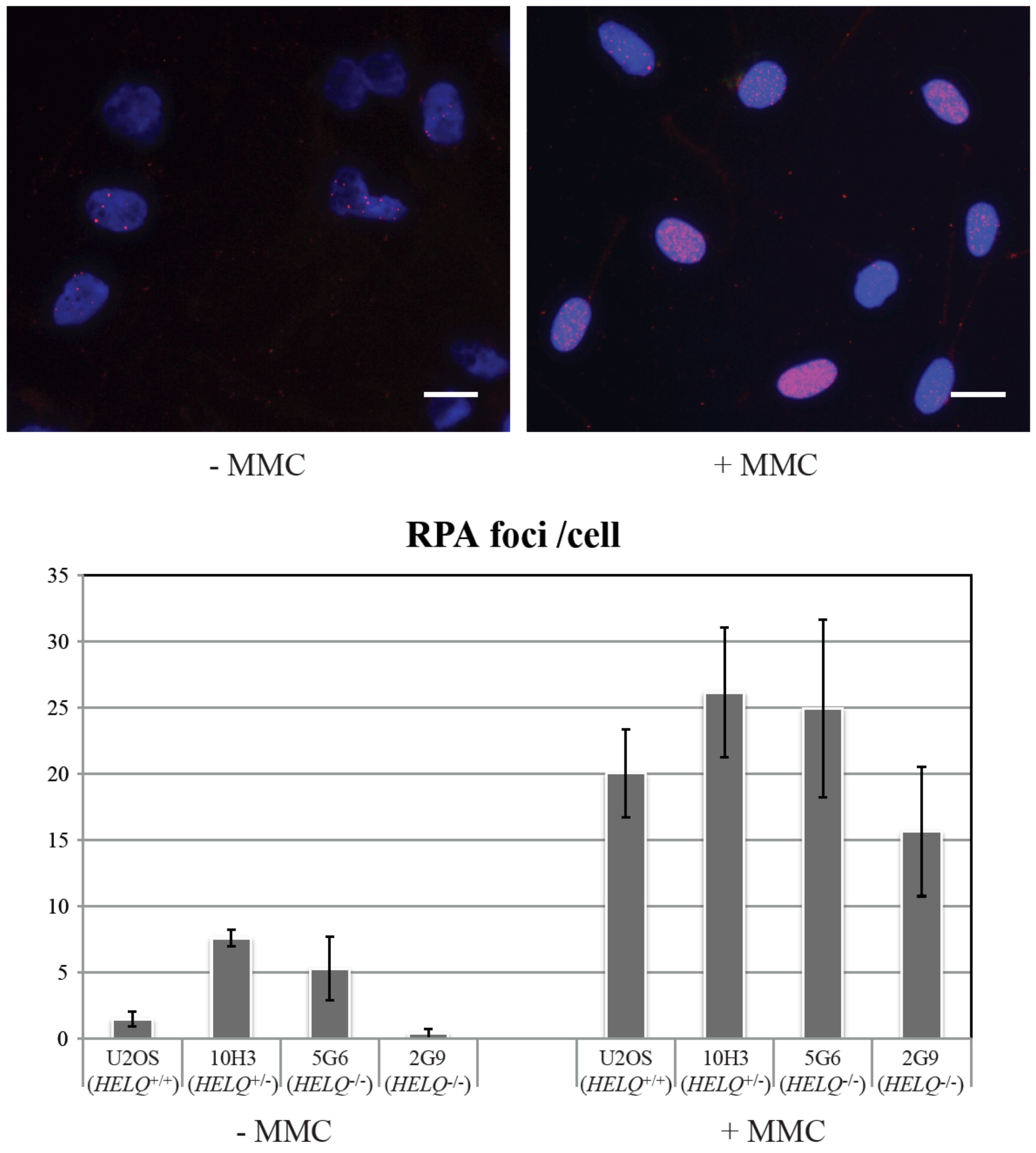

Supplementary Figure S4. **RAD51 and RPA foci formation in *HELQ* knockout cells.** Indicated cells were mock treated or treated with 100 ng/ml MMC for 24 hr. Nuclei are shown in blue, RAD51 foci in green (A), and RPA foci in red (B). The foci number per cell ± standard error from two biological replicates is shown. Scale bars: 20 μm.

Supplementary Figure S5

Figure 3A

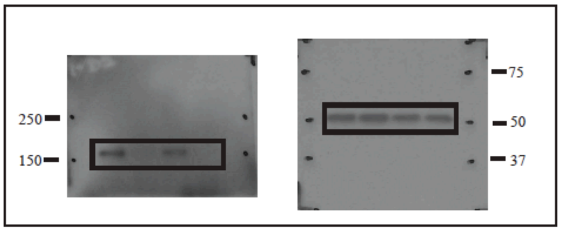

Figure 3B

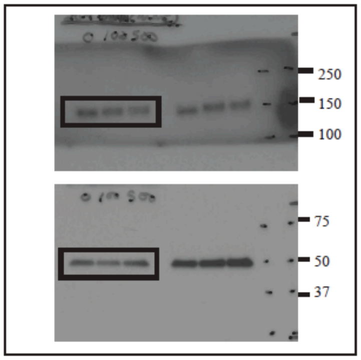

Figure 4B

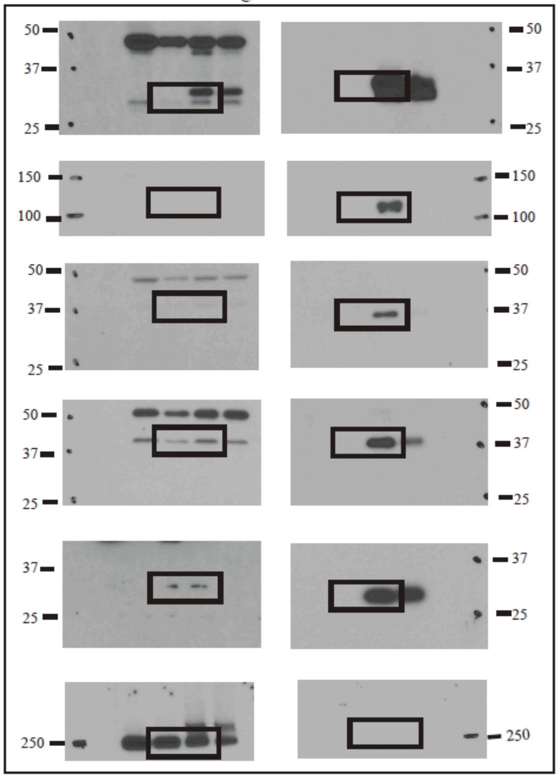

Figure 4A

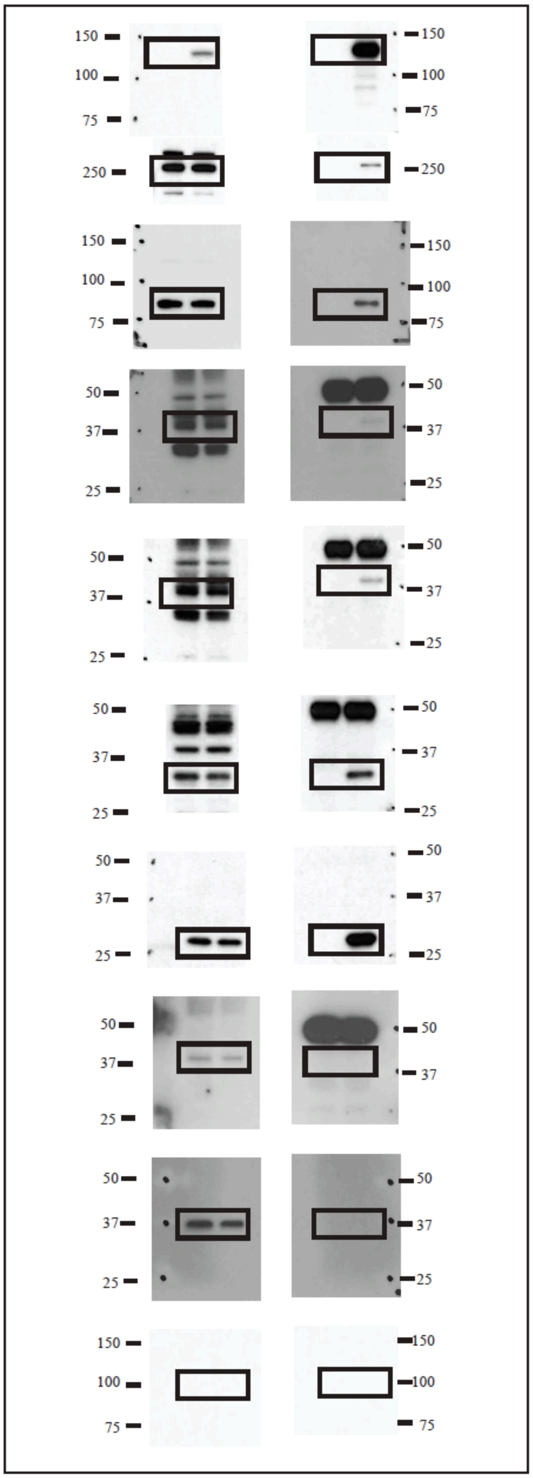

Supplementary Figure S5 (continued)

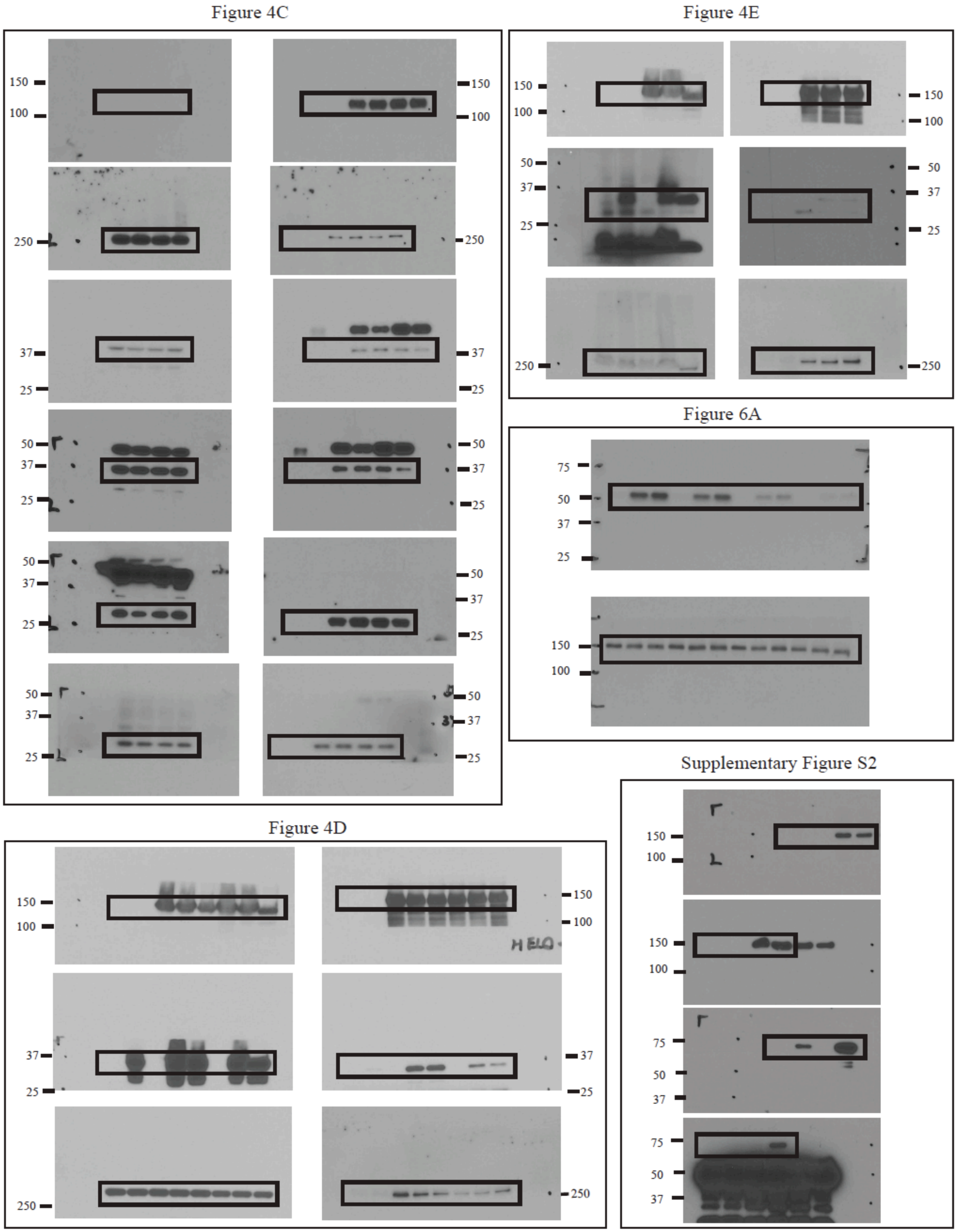

Supplementary Figure S5. Full length images of immunoblots.

Supplementary Table S1. Proteins identified in the HELQ complex by LC/MS/MS analysis*

Protein	Accession Number (UniprotKB/Swiss-Prot)	Molecular Weight	Spectral counts	Unique peptides
HELQ	Q8TDG4|HELQ_HUMAN	124 kDa	1228	133
ATR	Q13535|ATR_HUMAN	294 kDa	15	12
RAD51D	O75771|RA51D_HUMAN	35 kDa	17	10
RAD51B	O15315|RA51B_HUMAN	47 kDa	16	9
RAD51C	O43502|RA51C_HUMAN	42 kDa	11	6
XRCC2	O43543|XRCC2_HUMAN	32 kDa	8	3
ATRIP	Q8WXE1|ATRIP_HUMAN	83 kDa	6	5
XRCC3	O43542|XRCC3_HUMAN	38 kDa	0	0
RAD51	Q06609|RAD51_HUMAN	37 kDa	0	0
POLN	Q7Z5Q5|DPOLN_HUMAN	100 kDa	0	0
FANCD2	Q9BXW9|FACD2_HUMAN	166 kDa	0	0

Supplementary Table S2. Proteins identified in the XRCC2 complex by LC-MS/MS analysis

Protein	Accession Number (UniprotKB/Swiss-Prot)	Molecular Weight	Spectral counts	Unique peptides
XRCC2	O43543|XRCC2_HUMAN	32 kDa	548	25
HELQ	Q8TDG4|HELQ_HUMAN	124 kDa	134	47
RAD51D	O75771|RA51D_HUMAN	35 kDa	229	23
RAD51B	O15315|RA51B_HUMAN	38 kDa	167	22
RAD51C	O43502|RA51C_HUMAN	42 kDa	146	18
XRCC3	O43542|XRCC3_HUMAN	38 kDa	0	0
RAD51	Q06609|RAD51_HUMAN	37 kDa	0	0
ATR	Q13535|ATR_HUMAN	294 kDa	0	0
ATRIP	Q8WXE1|ATRIP_HUMAN	83 kDa	0	0
POLN	Q7Z5Q5|DPOLN_HUMAN	100 kDa	0	0

* Proteins from gel sections were identified by LC-MS/MS. The gel sections were approximately equal size except the HELQ, which was cut and run as a single band with longer run times. Proteins not identified in the complexes were not detected from at least two independent LC-MS/MS analyses.

SUPPLEMENTARY METHODS

Cell culture. U2OS (ATCC HTB-96) and HEK293T (ATCC CRL11268) cells were cultured in Dulbecco's Modified Eagle's Medium supplemented with 10% fetal bovine serum and 1% penicillin-streptomycin (Invitrogen) and maintained in a humidified 5% CO_2 incubator at 37 °C. HeLa S3 (ATCC CCL2.2) cells were cultured in RPMI 1640 medium supplemented with 10% fetal bovine serum and 1% penicillin-streptomycin. All cells were tested for mycoplasma infection and were negative. The identity of all cell lines including the zinc finger nuclease disruptants was confirmed using small tandem repeat (STR) DNA fingerprinting by the Characterized Cell Line Core of the M.D. Anderson Cancer Center.

Antibodies. Anti-HELQ (2406c, 1:1,000 dilution), anti-ATR (N-19, 1:1,000 dilution), anti-CHK1 (G-4, 1:1,000 dilution), and HRP (horseradish peroxidase) conjugated anti-goat IgG (sc-2033, 1:20,000 dilution) were purchased from Santa Cruz Biotechnology, Inc. Anti-ATRIP (A300-095A, 1:10,000 dilution) and anti-SMC1 (A303-834A, 1:2,000 dilution) were purchased from Bethyl Laboratories, Inc. Anti-RAD51C (2H11/6, 1:1,000 dilution) and anti-RAD51D (NB100-166, 1:1,000 dilution) were purchased from Novus Biologicals. Anti-FANCD2 (EPR2302, 1:2,000 dilution) was purchased from GeneTex Inc. Anti-RPA70 (ab79398, 1:5,000 dilution) and anti-Histone H3 (ab1791, 1:5,000 dilution) were purchased from Abcam. Anti-phospho Ser345 CHK1 (133D3, 1:1,000 dilution) was purchased from Cell Signaling Technology. Anti-RAD51B (1H3, 1:1,600 dilution) was purchased from AffinityBioReagents. Anti-α-tubulin (T5168, 1:8,000 dilution), HRP conjugated anti-mouse IgG (A0168, 1:20,000 dilution), HRP conjugated anti-rabbit IgG (A0545, 1:20,000 dilution), anti-FLAG M2 (F3165, 1:10,000 dilution), and anti-FLAG M2 affinity agarose gel (A2220) were purchased from Sigma-Aldrich. Anti- RAD51 (B01P, 1:1,000) was purchased from Abnova. Anti-GFP (RQ2) agarose was purchased from MBL International Corporation. Anti-XRCC2 (SWE35, 1:6,000 dilution), anti-XRCC3 (10F1, 1:2,000 dilution), and anti-RAD51 (FBE2, 1:4,000 dilution) were gifts from Stephen C. West (Cancer Research UK). Anti-POLN (PA434, 1:1,000 dilution) was raised against purified recombinant POLN [22,61].

Plasmid Constructs. Full length wild type or HELQ K365M open reading frame (ORF) sequences [16] were PCR amplified as a SalI-NotI fragment with 5'HELQ (SalI) primer (5'-CACCGTCGACATGGATGAATGTGGTTCCCGCATCCG) and 3'HELQ (NotI) primer (5'-GCGGCCGCTCATGCTTTGTCAGTGGAAGAAGCCACAGC) to clone into pOZN[21] or pETDuet-1 (Novagen). The SalI-EcoRV fragments from WT or K365M HELQ/pETDuet-1 were inserted into pEGFP-C1 (Clontech) to generate HELQ/pEGFP-C1 and K365M HELQ/pEGFP-C1. Full length wild type XRCC2 ORF sequences were PCR amplified as a SalI-NotI fragment with 5'XRCC2 (SalI) primer (5'-CACCGTCGACTGACACCATGTGTAGTGCCTTCCA-TAGGGCTGAGTC) and 3' XRCC2 (NotI) primer (5'-TAAAAGCGGCCGCACAAAATTC-AACCCCACTTTCTCC) and cloned into pOZC[21]. To generate RAD51B/pDsRed-Monomer-

Hyg-C1, RAD51B ORF was PCR amplified as a XhoI-SmaI fragment with 5'RAD51B (XhoI) primer (5'-CCGCTCGAGATGGGTAGCAAGAAACTAAAACGAGTGGG) and 3' RAD51B (SmaI) primer (5'-TAAAACCCGGGTTAAAAAATTAGCTGGGTATGGTGGCACATCTG) to clone into pDsRed-Monomer-Hyg-C1 (Clontech).

Mass Spectrometry. Proteins were identified by LC-MS/MS using either the Proxeon Easy-nLC II or the Dionex Ultimate 3000 RSLCnano LC coupled to the Thermo Velos Pro or the Orbitrap Elite. Prior to HPLC separation, the peptides were desalted using Millipore U-C18 ZipTip Pipette Tips following the manufacturer's protocol. A 2 cm long x 100 μm I.D. C18 5 μm trap column (Proxeon EASY Column) was followed by a 75 μm I.D. x 15 cm long analytical column packed with C18 3 μm material (Dionex Acclaim PepMap 100). Buffer A was composed of 0.1% formic acid in water and Buffer B 0.1% formic acid in acetonitrile. For most samples, data was acquired for 35 min using an HPLC gradient of 5% B to 45% B over 30 min with a flow rate of 300 nl/min. For the HELQ band, the acquisition time was 65 min, and the HPLC gradient was 5% B to 8% B at 5 min, to 27% B at 39 min, and to 45% B at 54 min. For Velos Pro acquisition, the data dependent method consisted of an MS scan followed by CID MS/MS of the top 10 precursor ions. For Elite samples (XRCC2 gel sections), the FT-MS resolution was set to 120,000, and top 20 MS/MS were acquired in CID ion trap mode. For the HELQ band, FT-MS at 120,000 resolution was followed by FT-MS/MS at 15,000 resolution in the CID mode of the top 10 precursor ions. Raw data was processed using SEQUEST version 1.3.0.339 embedded in Proteome Discoverer v1.3 using the following parameters: full trypsin digest with maximum 2 missed cleavages, fixed modification carbamidomethylation of cysteine, variable modification oxidation of methionine, searching the human reference proteome from Uniprot dated January 22, 2013 with 87,450 entries. The mass accuracy was set to 1.2 Da average mass for Velos Pro and 10 ppm monoisotopic mass for Elite FT-MS experiments. The accuracy for fragment ions was 0.8 Da monoisotopic for ion trap MS/MS and 0.02 Da for FT-MS/MS. A decoy database was generated from the Uniprot human database and used by Peptide Validator and Scaffold for calculating false discovery rates. X!Tandem (The GPM, version CYCLONE (2010.12.01.1)) database searches were performed embedded in Scaffold Q+ version 3.6.4 (Proteome Software) using the same search parameters as SEQUEST. Scaffold was used for validation of peptide identifications with confidence filtering for peptides of greater than 95% probability based on the Peptide Prophet algorithm. Protein identification established greater than 99.9% protein probability assigned by the Protein Prophet algorithm, with a minimum of 2 peptides at 95% peptide probability. Peptide and protein false discovery rates were calculated as 0.0% by Scaffold. Abundant proteins found commonly in immunoprecipitation experiments with these epitope tags were eliminated from consideration [62-65]. Protein identifications were checked for agreement with the molecular mass predicted from the relevant gel slice.

Immunofluorescence. 2 x 10^4 cells were plated on each well of 4-well chamber slides. At 24hr the cells were treated with or without 100 ng/ml of mitomycin C (MMC). At 24 hr post MMC

treatment, the cells were fixed with 4% formaldehyde in PBS for 10 min at room temperature and permeabilized with 0.1% NP40 in PBS for 10 min at room temperature. The cells were incubated in blocking buffer (TBS buffer containing 10% donkey serum and 0.01% Tween 20) for 30 min at room temperature, and incubated with primary antibodies against RAD51 (mouse monoclonal, 1:1,000, Abnova). The preparation was incubated with AlexaFluor-488 goat-anti-mouse antibody (1:3000, Invitrogen), incubated with DAPI in PBS, and mounted with VectaShield mounting medium. The cells were analyzed using a Leica DMI6000 microscope, with 40X and quantification was done with ImageJ. To analyze chromatin bound RPA70, the cells were permeabilized in cytoskeleton (CSK) buffer (10 mM PIPES (pH 7.0), 100 mM NaCl, 300 mM sucrose, 3 mM $MgCl_2$, 1 mM EGTA, 10 mM NaF, 1.35% β-glycerophosphate, 0.2% Triton X-100, freshly supplemented with 0.275 mM ATP, and protease inhibitors (Roche Applied Science)) for 5 min on ice, incubate in cytoskeletal stripping buffer (10 mM Tris, pH 7.4; 10 mM NaCl; 3 mM $MgCl_2$; 1% Tween 20; 0.5% Na deoxycholate) for 5 min on ice, washed 3 times with PBS, and fixed in 2% formaldehyde-2% sucrose in PBS for 10 min. RPA70 was visualized RPA70 (rabbit monoclonal, 1:500, abcam) and AlexaFluor-594 goat-anti-rabbit secondary antibody (1:1,600, Invitrogen). At least 50 cells per sample were analyzed to identify the ratio of foci.

Radial chromosome analysis. Cells were treated with 0, 15 or 25 ng/ml of MMC for 48 hr. At forty-four hr post MMC treatment, cells were treated with 14.6 ng/ml colcemid solution (Sigma) for 4 hr. The cells were then trypsinized and exposed to 0.075 M KCl for 15 min at 37°C, and were fixed in 3:1 methanol:glacial acetic acid. The cells were spread on glass slides, Giemsa stained and metaphases were analyzed using a BX41 Olympus microscope, with 60X or 100X oil objectives. Photographs were taken with the 60X oil objective on a Spot Idea 5 color digital camera. 150 metaphases per sample were analyzed to identify cell population with radial chromosome.

SUPPLEMENTARY REFERENCES

61 Marini, F., Kim, N., Schuffert, A. & Wood, R. D. POLN, a nuclear PolA family DNA polymerase homologous to the DNA cross-link sensitivity protein Mus308. *J Biol Chem* **278**, 32014-32019 (2003).

62 Jung, S. Y., Malovannaya, A., Wei, J., O'Malley, B. W. & Qin, J. Proteomic analysis of steady-state nuclear hormone receptor coactivator complexes. *Mol Endocrinol* **19**, 2451-2465 (2005).

63 Chen, G. I. & Gingras, A. C. Affinity-purification mass spectrometry (AP-MS) of serine/threonine phosphatases. *Methods* **42**, 298-305 (2007).

64 Trinkle-Mulcahy, L. *et al.* Identifying specific protein interaction partners using quantitative mass spectrometry and bead proteomes. *J Cell Biol* **183**, 223-239 (2008).

65 Guo, R., Xu, D. & Wang, W. Identification and analysis of new proteins involved in the DNA damage response network of Fanconi anemia and Bloom syndrome. *Methods* **48**, 72-79 (2009).
